# Supplementary material for: The Effect of DBD Plasma Activation Time on the Dyeability of Woven Polyester Fabric with Disperse Dye
Source: Polymers (Basel). 2021 Apr 29;13(9):1434. doi: 10.3390/polym13091434 (PMC8125205; doi:10.3390/polym13091434)
Supplement: Supplementary file 1 [file polymers-13-01434-s001.zip › polymers-1145897-supplementary.pdf]

Supplementary Materials

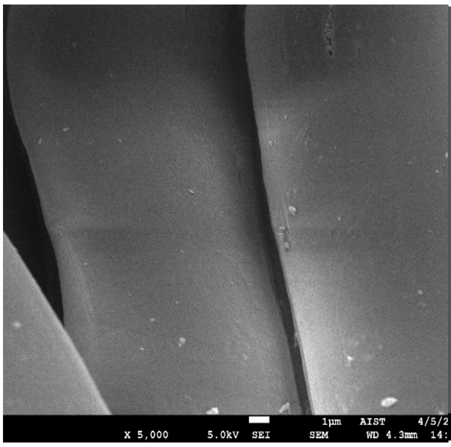

(**a**) P0′ × 5000

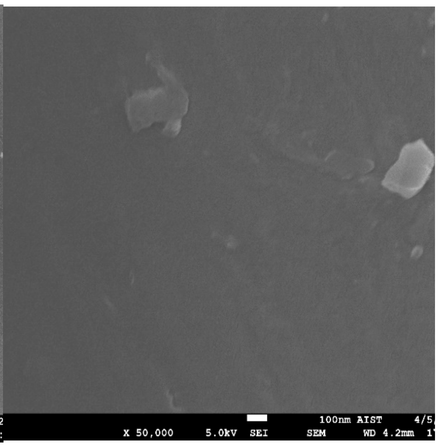

(**b**) P0′ × 50000

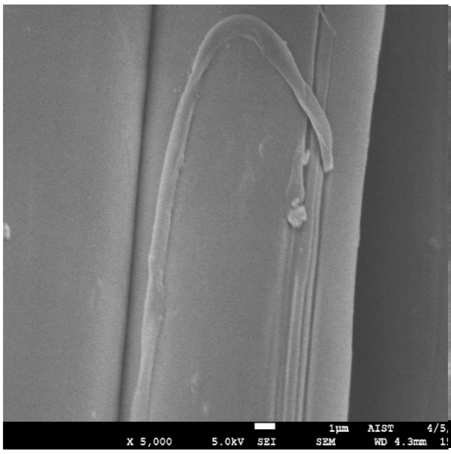

(**c**) P30′ × 5000

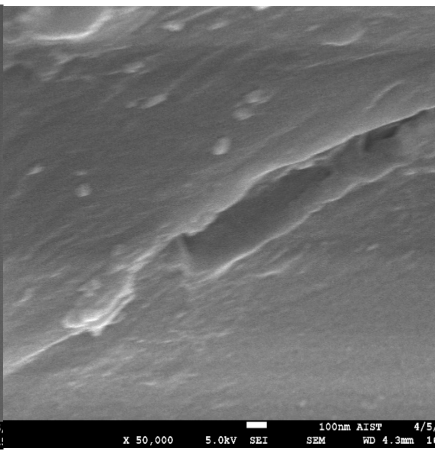

(**d**) P30′ × 50000

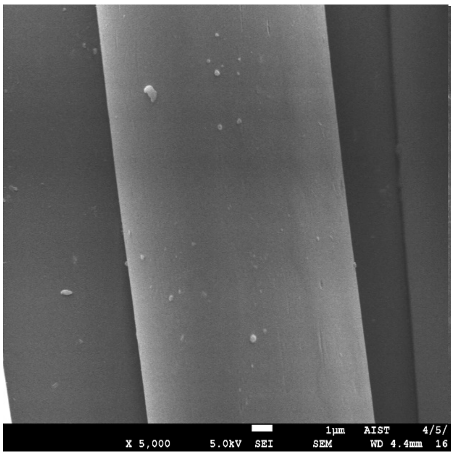

(**e**) P60′ × 5000

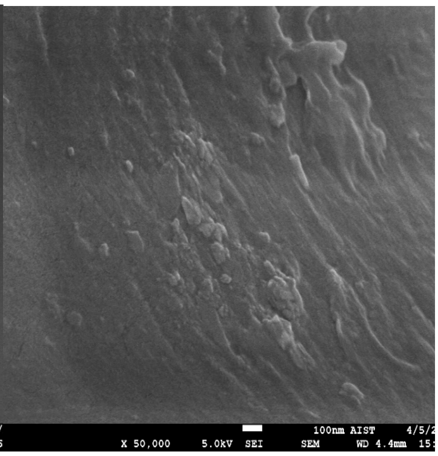

(**f**) P60′ × 50000

Figure S1. SEM images of the polyester fibers after 1 rinsing cycle with acetone: (**a**,**b**) untreated polyester, (**c**,**d**) plasma-treated polyester for 30 s, (**e**,**f**) plasma-treated polyester for 60 s.
